# Presumed sarcoid choroiditis: referral pathways, demographic characteristics and disease phenotypes

**DOI:** 10.1186/s12348-026-00595-w

**Published:** 2026-06-03

**Authors:** Supawat Trepatchayakorn, Carolina Bernal-Morales, Cristina Arpa, Jose Carlo M. Artiaga, Harry Petrushkin

**Affiliations:** 1 Moorfields Eye Hospital NHS Foundation Trust, City Road, London, EC1V 2PD UK; 2 Hospital Clínic de Barcelona, Institut Clínic d’Oftalmologia, Barcelona, Spain; 3 Philippine Eye Research Institute, National Institutes of Health, University of the Philippines Manila, Manila City, Philippines; 4 Department of Ophthalmology and Visual Sciences, Philippine General Hospital, University of the Philippines Manila, Manila City, Philippines; 5 Great Ormond Street Hospital, Great Ormond Street, London, WC1N 3JH UK

## Abstract

**Purpose:**

To audit the current clinical process whereby patients with presumed sarcoid choroiditis are referred for systemic investigation. To describe demographic and phenotypic heterogeneity of patients clinically diagnosed with presumed sarcoid choroiditis, and to compare between those who were concordant and non-concordant to the 2019 International Workshop on Ocular Sarcoidosis (IWOS) diagnostic criteria.

**Method:**

Retrospective audit of patients clinically diagnosed with presumed sarcoid choroiditis evidenced by the presence of hypo-fluorescent dark dots (HDDs) on indocyanine green angiography (ICGA). Cases with serologic evidence of tuberculosis were excluded. A target of six months was set between diagnosis and referral to a sarcoidosis clinic. Demographic data, intra-ocular clinical signs, and systemic investigations—as outlined by IWOS criteria—were analysed and compared between IWOS-concordant and IWOS-non-concordant groups.

**Results:**

Sixty-one cases of suspected sarcoid choroiditis based on clinical diagnosis plus HDDs on ICGA and negative serological tests for tuberculosis were included. Following referral, 56.8% of patients were systemically investigated by a sarcoidosis specialist within six months. Mean age was 46.6 ± 17.1 years, 54.1% were male, and 44.3% Caucasian. 72% (72%) of eyes fulfilled IWOS diagnostic criteria, while 28% did not. Patients who met IWOS criteria were significantly more likely to have a systemic diagnosis of sarcoidosis versus the IWOS non-concordant group (41.2% vs. 0%, *p* = 0.010).

**Conclusion:**

Approximately half of patients with presumed sarcoid choroiditis were referred for systemic evaluation within 6 months. Indocyanine green angiography (ICGA) is a useful diagnostic tool in the diagnosis of sarcoid choroiditis. ICGA may help identify systemic sarcoidosis in those who do not meet the IWOS criteria.

**Supplementary Information:**

The online version contains supplementary material available at 10.1186/s12348-026-00595-w.

**Table:** 

**SUMMARY BOX**	
*What was known before:*	
• Ocular sarcoidosis may be one of the initial presentations of systemic sarcoidosis prior to manifestations in other organs.	
• The 2019 International Working on Ocular Sarcoidosis Criteria is a helpful tool in diagnosing most cases of ocular sarcoidosis, especially in the absence of definitive biopsy results.	
*What this study adds:*	
• Only roughly half of patients are currently being referred for systemic evaluation and a third being evaluated within six months.	
• Indocyanine green angiography may help identify sarcoidosis which would otherwise be undiagnosed with present consensus criteria.	

## Introduction

Ocular sarcoidosis is one of the leading causes of sight-threatening uveitis, yet it still poses a diagnostic challenge due to its varied presentation [1]. Gold standard for diagnosis of sarcoidosis is histopathological identification of non-caseating granulomas with the exclusion of other infective causes, most notably tuberculosis. Due to difficulties in obtaining definitive biopsy results, diagnostic criteria have been proposed by the International Workshop on Ocular Sarcoidosis (IWOS), comprising of intra-ocular features and systemic investigative results [2, 3]. Moreover, 26% of patients with systemic sarcoidosis have been reported to manifest ocular abnormalities, suggesting a substantial association between ocular and systemic diseases [4, 5].

Indocyanine green angiography (ICGA) is an increasingly useful diagnostic tool to evaluate choroidal involvement in ocular sarcoidosis [6]. Active sarcoid choroiditis usually results in hypo-fluorescent dark dots (HDDs) which are present in the intermediate phase (8–10 min) and fade in the late phase (28–32 min), indicating that an active inflammatory process may lie deep in the choroidal stroma with relative preservation of the choriocapillaris, not unlike those seen in Vogt-Koyanagi-Harada (VKH) or sympathetic ophthalmia (SO), although they are typically less evenly sized and more randomly distributed [7]. As ICGA is not yet incorporated into the 2019 IWOS diagnostic criteria, there is limited understanding of clinical phenotypes—ocular and systemic—among a subgroup of patients with these ICGA abnormalities.

This is the first in an audit cycle aiming to optimise and harmonise referral pathways for this group of patients. This paper investigates the timeliness of systemic evaluation following referral from our Uveitis clinic upon suspicion of sarcoidosis. In order to understand the heterogeneity of clinical phenotypes to help in the creation of formal guidelines, we describe the demographic profile, ocular signs, and systemic investigation results of patients who were clinically diagnosed with sarcoid choroiditis with positive ICGA findings, and compare the presence of each IWOS-defined item amongst eyes which are concordant and those which are non-concordant to the 2019 IWOS diagnostic criteria for ocular sarcoidosis.

## Material & method

A retrospective audit was conducted in a single, high-volume uveitis referral centre in London, United Kingdom, and approved by the Institutional Clinical Audit Board (audit number CA22/UV/1007). Electronic hospital records were searched for patient correspondence letters on OpenEyes (The Apperta Foundation CIC, England) between March 2012 and March 2022 via Magic XPA v3.3 (Magic Software Enterprises, Israel) tool. The key words ‘sarcoid’ and ‘choroid’ were used with proper Boolean operators. Physical hospital records from our institution were retrieved for all identified patients to collect data not available from the electronic database.

**Fig. 1:**
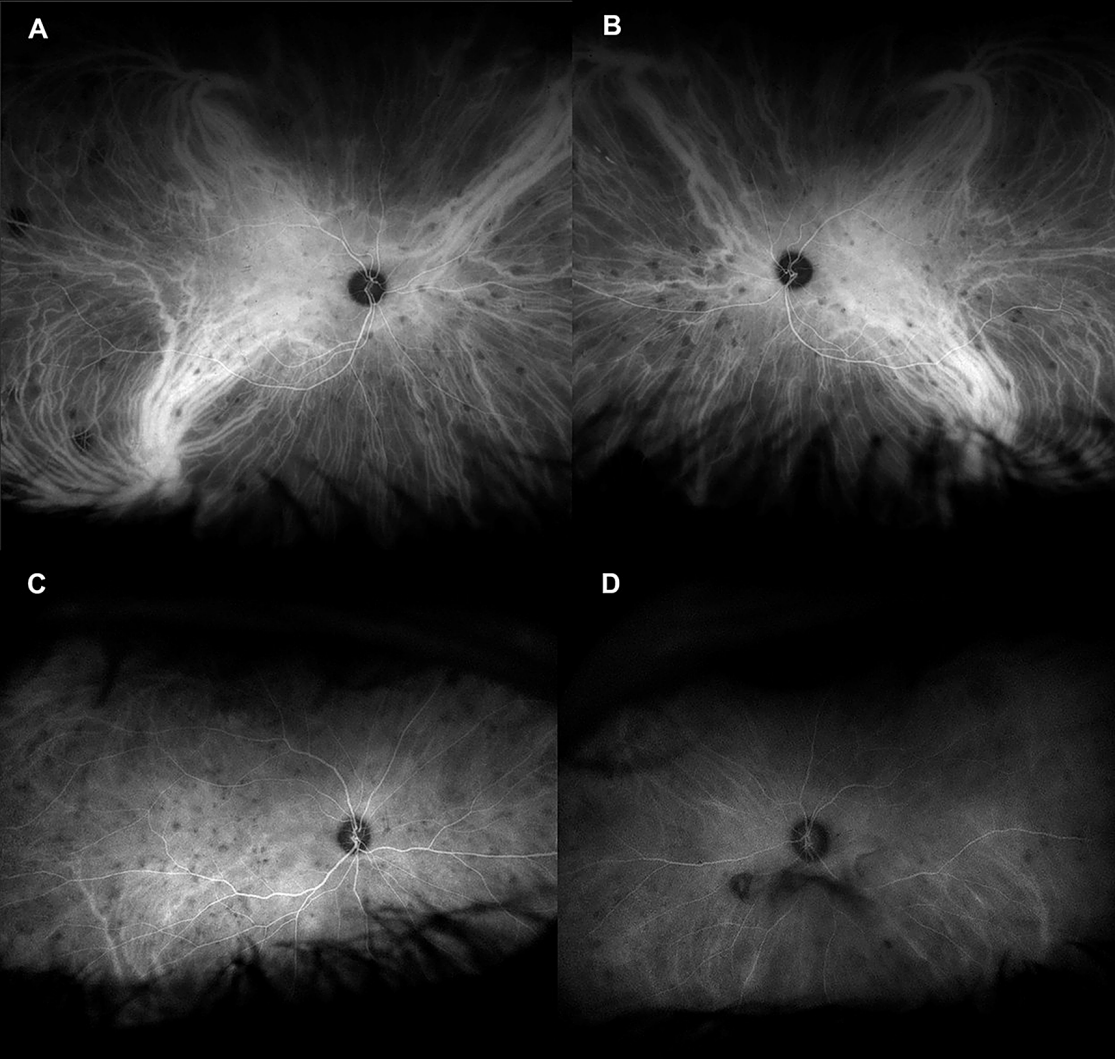
Representative images of hypofluorescent dark dots (HDDs) on indocyanine green angiography (ICGA): (**A** and **B**) in an IWOS-concordant patient and (**C** and **D**) in an IWOS-nonconcordant patient. IWOS = International Workshop on Ocular Sarcoidosis

Patients with both a clinical diagnosis of ocular sarcoidosis and the presence of irregularly distributed HDDs in the intermediate phase (8–10 min) of ICGA, which may or may not fade in the late phase (28–32 min), were included (Fig. 1). Uveitis screening blood tests—including, but not limited to, syphilis IgG, HIV, toxoplasma IgG, ANA, ENA, anti-dsDNA, and ANCA—were done and yielded negative results in all cases. Exclusion criteria include positive interferon-γ release assay (IGRA) or a different diagnosis on subsequent visits based on clinical and/or diagnostic findings.

Time points when the patient was reviewed by a respiratory specialist (‘referral-to-review time’) was determined by scrutinising all subsequent correspondence letters following the decision to refer from the Uveitis clinic. A referral was considered “timely” if the referral-to-review time did not exceed six months— a target pre-specified by the uveitis service and sarcoidosis teams.

Baseline demographic data was collected for each patient and included age at presentation, gender, ethnicity, and laterality. Presence or absence of intra-ocular clinical signs and positive systemic investigation results as outlined by the 2019 IWOS criteria were noted (see Appendix 1). Intra-ocular signs included; mutton-fat keratic precipitates (KPs) or iris nodules (Koeppe/Busacca), trabecular meshwork (TM) nodules or tent-shaped peripheral anterior synechiae (PAS), snowballs or string of pearls vitreous opacities, chorioretinal peripheral lesions— both active and inactive, peri-phlebitis or macro aneurysm, and optic disc or choroidal granuloma.

Systemic investigations recorded were mostly derived from the IWOS and the American Thoracic Society (ATS) recommended items. These included bilateral hilar lymphadenopathy (BHL) either on chest x-ray (CXR) or pulmonary CT scan; abnormal serum level of angiotensin converting enzyme (ACE), soluble IL-2 receptors, calcium, creatinine, liver function test (LFTs)— which include aspartate and alanine aminotransaminases (AST and ALT), and alkaline phosphatase (ALP); lymphopaenia; and endobronchial ultrasound (EBUS) or mediastinoscopic lymph node sampling results [2, 4]. Diagnoses of systemic sarcoidosis syndrome (Löfgren, Heerfordt, or lupus pernio) were also recorded, if present. Patients were then further classified as either ‘IWOS-concordant’ should one of the three IWOS categories’ (probable, presumed, or definite ocular sarcoidosis) diagnostic criteria were fulfilled, or ‘IWOS-non-concordant’ otherwise. In case of incomplete data, no missing values were substituted.

## Statistical analysis

Descriptive statistical analysis was performed using the Excel tool (Microsoft Corporation, Washington, USA). Descriptive statistics were performed using frequency statistics for qualitative variables. Continuous quantitative variables were expressed as mean ± standard deviation (SD). Differences between qualitative categorical variables were assessed with the chi-squared (χ2) test, or the Fisher’s exact test in case of small-size samples [8]. Normality of quantitative variables was evaluated with the Shapiro-Wilk and the Kolmogorov-Smirnov tests. The paired t-test was used to compare quantitative variables if distributed normally, and the Mann-Whitney U test was performed when non-parametric tests were required. A p-value of < 0.05 was considered statistically significant. Comparative statistical analysis was performed with the SPSS 25.0 software (IBM SPSS Statistics v25.0, Armonk, NY, IBM Corp).

## Results

A total of 61 cases were eligible for final analysis. Forty-four cases (72.1%) fulfilled one of the three IWOS categories—probable (14.8%), presumed (13.1%), or definite (44.3%) ocular sarcoidosis—while 17 cases (27.9%) did not, but were considered to have clinical signs suggestive of ocular sarcoidosis.

Mean age was 46.6 ± 17.1 years (47.0 ± 16.1 in the IWOS-concordant group and 45.0 ± 19.9 in the IWOS-non-concordant group). Male patients made up to 54.1% of the overall cohort (56.8% in IWOS-concordant group and 47.1% in IWOS-non-concordant group). Bilateral disease was found in 90.2% of overall cases (90.9% in IWOS-concordant group and 88.2% in IWOS-non-concordant group). None of the differences between groups were statistically significant. Regarding ethnicity, 44.3% of patients were Caucasian, 14.7% African, and 9.8% Asian; 13.1% identified with other ethnicities and in 18% ethnicity was unknown.

### Referral-to-review time

Twelve patients were known cases of systemic sarcoidosis and hence did not require a referral; three cases were referred but the referral-to-review time could not be discerned, while data was missing for another nine patients. Among those that could be ascertained (*n* = 37), referral-to-review time was within six months in 56.8% (21/37) of patients.

### Intra-ocular signs

Mutton-fat KPs and/or iris nodules were found in 37.7% (23/61) of cases (43.2% in IWOS-concordant group and 23.5% in IWOS-non-concordant group). TM nodules and/or tent-shaped PAS were documented in 6.6% (4/61) of cases (4.5% in IWOS-concordant group and 11.8% in IWOS-non-concordant group).

Snowballs or string of pearls vitreous opacities were found in 60.7% (37/61) of cases (65.9% in IWOS-concordant group and 47.1% in IWOS-non-concordant group).

Multiple chorioretinal peripheral lesions were found in 85.3% (52/61) of cases (86.4% in IWOS-concordant group and 82.4% in IWOS-non-concordant group). The IWOS criterion of having nodular/segmental peri-phlebitis and/or macro aneurysm was fulfilled in 37.7% (23/61) of cases (38.6% in IWOS-concordant group and 35.3% in IWOS-non-concordant group).

Of note, the IWOS criterion of having optic disc nodules/granuloma and/or solitary choroidal granuloma was fulfilled in 14.8% of cases. This was notably higher (29.4%) in the IWOS-non-concordant group than the IWOS-concordant group (9.1%); the difference, though, remained statistically insignificant between the two groups (Table 1).

**Table 1:** Intra-ocular clinical signs

	IWOS-concordant (*n* = 44)	IWOS non-concordant (*n* = 17)	Total (*n* = 61)	*p* value
Mutton-fat KPs and/or iris nodules	43.2% (19)	23.5% (4)	37.7% (23)	0.239
TM nodules *and/or* tent-shaped PAS	4.5% (2)	11.8% (2)	6.6% (4)	0.308
Snowballs/ string of pearls	65.9% (29)	47.1% (8)	60.7% (37)	0.244
Multiple chorio-retinal peripheral lesions	86.4% (38)	82.4% (14)	85.3% (52)	0.699
Nodular/segmental peri-phlebitis *and/or* macro aneurysm	38.6% (17)	35.3% (6)	37.7% (23)	1.000
Optic disc nodules/granulomas *and/or* solitary choroidal nodule	9.1% (4)	29.4% (5)	14.8% (9)	0.100

### Systemic investigations

#### IWOS-defined items

Bilateral hilar lymphadenopathy (BHL) was documented to be present on either chest x-ray (CXR) or CT scans in 40.4% of cases (54.6% in IWOS-concordant group and 7.1% in IWOS-non-concordant group). Serum ACE level was raised (> 52 IU/L) in 56.1% of cases (65.0% in IWOS-concordant group and 35.3% in IWOS-non-concordant group). Lymphopaenia (≤ 1.2 × 10⁹/L) was present in 27.8% of cases (37.8% in IWOS-concordant group and 5.9% in IWOS-non-concordant group). Differences between groups were statistically significant for all these characteristics (see Table 2).

Serum lysozyme, bronchoalveolar lavage (BAL) fluid CD4:CD8 ratio, and Gallium^67^ scintigraphy or 18 F-fluorodeoxyglucose positron emission tomography (PET) scans, while included in the IWOS criteria, were not requested in any of the cases in this study and hence were not reported.

**Table 2:** Systemic investigations

	IWOS-concordant	IWOS-non-concordant	Total	*p* value
**IWOS-defined items**				
BHL on CXR or CT scan	54.6% (18/33)	7.1% (1/14)	40.4% (19/47)	0.003
↑ Serum ACE	65.0% (26/40)	35.3% (6/17)	56.1% (32/57)	0.047
Lymphopaenia	37.8% (14/37)	5.9% (1/17)	27.8% (15/54)	0.021
**Non-IWOS defined items**				
↑ Serum IL-2R	50.0% (2/4)	33.3% (2/6)	40.0% (4/10)	1.000
↑ Serum calcium	8.3% (1/12)	0.0% (0/4)	6.25% (1/16)	1.000
Abnormal LFTs (AST, ALT, or ALP)	36.8% (14/38)	0.0% (0/16)	25.9% (14/54)	0.005
↑ Serum creatinine	16.7% (6/36)	5.9% (1/17)	13.2 (7/53)	0.408
EBUS/mediastinoscopic lymph node sampling	70.8% (17/24)	0.0% (0/4)	60.7% (17/28)	0.016
Systemic involvement (pulmonary, other organs)	41.2% (14/34)	0.0% (0/11)	31.1% (14/45)	0.010

## Discussion

This audit evaluated the timeliness of referrals for presumed sarcoid choroiditis against our internally defined gold-standard target of six months. We also characterised the demographic, ocular, and systemic features of the cohort, based on clinical suspicion and presence of HDDs on ICGA. In addition, we report the prevalence of key intraocular signs and systemic investigations and compare findings between the IWOS-concordant and IWOS-non-concordant subgroups. These results can be considered alongside previously published ocular and systemic sarcoidosis cohorts.

Sarcoidosis is a multi-system inflammatory disease that can affect multiple organs and cause serious, potentially life-threatening complications—particularly pulmonary and cardiac—if not detected and treated early [4]. Early work by Jabs et al. reported ocular involvement in up to 25% of patients with sarcoidosis, with uveitis often representing a common and early manifestation [5]. More recently, Ma et al. found that 80% of patients in their cohort initially presented with uveitis symptoms, and that 43.9% subsequently developed new systemic organ involvement within 12 months [9]. These findings underscore the importance of timely referral for systemic evaluation following a diagnosis of ocular sarcoidosis. In our audit, only 56.8% of patients were evaluated within the pre-specified six-month referral window, highlighting a clear area for service improvement and a potential risk of delayed detection systemic disease.

Within this cohort, 19.7% of patients had a pre-existing diagnosis of systemic sarcoidosis and therefore did not require further referral. However, as this audit was conducted within a specialist ophthalmic hospital, access to wider medical records was limited. Consequently, we were unable to assess the impact of referral delays on systemic disease outcomes, representing an important area for future audit and service evaluation.

Awareness of the diverse manifestations of ocular sarcoidosis is essential for ophthalmologists. The International Workshop on Ocular Sarcoidosis (IWOS) criteria, most recently revised in 2018 and published in 2019, have played a central role in standardising the diagnosis of ocular sarcoidosis [2]. Applying these criteria, Acharya et al. reported that 23% of 884 patients with ocular sarcoidosis were of white ethnicity in a multinational study spanning 19 centres across 12 countries [10]. In contrast, 44% of patients in our cohort identified as Caucasian, representing a substantially higher proportion. This difference may partly be explained by the geographical location of our centre in central London, where 44.9% of the population identified as white as per the 2011 census [11]. In addition, all patients in our audit demonstrated posterior segment manifestations, which has been reported to be more prevalent in white individuals in several studies [12, 13]. Although all cases were assessed by uveitis specialists and considered phenotypically consistent with sarcoidosis rather than birdshot chorioretinitis (BSC), not all patients warrant or had negative HLA-A29 [14, 15].

The same study described the sensitivity of each of the IWOS-defined intra-ocular clinical signs and the results were comparable to our study for bilaterality (86% v 90%), snowballs/string of pearls (50% v 61%), mutton-fat KPs and/or iris nodules (46% v 38%), and nodular and/or segmental peri-phlebitis and/or macroaneurysm (37% v 38%) [10]. These findings suggest that the intraocular features observed in patients with sarcoid choroiditis may not differ substantially from those reported in broader ocular sarcoidosis cohorts. However, we observe a higher prevalence of optic disc nodules/granulomas and/or solitary choroidal nodule (15% v 7%) as well as multiple chorioretinal peripheral lesions (85% v 45%). This is unsurprising, as choroidal inflammation was a key inclusion criterion for this audit. In contrast, trabecular meshwork nodules and/or tent-shaped PAS were markedly less common in our cohort (6% v 39%). This may reflect a disease phenotype with predominant posterior segment involvement or alternatively may be confounded by the fact that gonioscopy is not routinely performed in our clinics.

Systemic investigations are central in the diagnosis of sarcoidosis, as the disease is predominantly systemic, with prominent pulmonary involvement up to 90% of cases [4]. The diagnosis of systemic sarcoid diagnosis is based on three key criteria: a compatible clinical presentation, histological confirmation of non-necrotising granuloma, and exclusion of alternative causes of granulomatous disease [3]. Reflecting this, the International Workshop on Ocular Sarcoidosis (IWOS) has placed increasing emphasis on systemic investigation findings within its diagnostic framework, expanding the number of suggestive systemic results from five to eight in its most recent revision [2]. This underscores the importance of timely respiratory referral for comprehensive systemic evaluation and appropriate management.

Among IWOS-inclusive criteria, our audit identified rates of lymphopænia (28%) and raised serum ACE level (56%) comparable to those reported in previous ocular and systemic sarcoidosis cohorts [4, 10, 16].

Pulmonary involvement is the most common extra-ocular manifestation of ocular sarcoidosis [17]. Bilateral hilar lymphadenopathy (BHL) on chest x-ray (CXR) is reported in over 90% in patients with systemic sarcoidosis [18–20]; however, lower rates have been reported in ocular disease. Acharya et al. reported positive BHL on CXR in 67% of ocular sarcoidosis cases, closer to the prevalence observed in our study (40%), and substantially lower than in systemic sarcoidosis [10, 20]. This may reflect isolated ocular involvement, or the protean, temporally evolving nature of sarcoidosis. Chest CT is more sensitive than CXR for detecting of BHL (94% to 64%) and should be considered in clinically suspected cases [21]. Further studies are needed to determine whether chest CT sensitivity differs between choroidal, ocular, and systemic sarcoidosis.

Abnormal hepatic and/or renal function test results were slightly more common in our cohort, possibly reflecting adverse effects of systemic immunotherapy initiated elsewhere, although this could not be confirmed due to limited access to systemic records. Serum calcium levels were comparable to previous reports, while the lower prevalence of raised serum IL-2R level may reflect limited testing. As IL-2R level testing becomes more widely used in uveitis practice, its diagnostic sensitivity and optimal cut-off value in sarcoid uveitis warrant further investigation [22].

Among definite ocular sarcoidosis (biopsy-proven with compatible uveitis) patients in this cohort (*n* = 27), 14 patients (51.9%) would not have otherwise fulfilled IWOS criteria for either presumed (2/27 with BHL) or probable (12/27 without BHL) ocular sarcoidosis without biopsy result despite having HDDs on ICGA. It should be cautioned, though, that serum lysozyme, bronchoalveolar lavage (BAL) fluid CD4:CD8 ratio, and Gallium^67^ scintigraphy or 18 F-fluorodeoxyglucose positron emission tomography (PET) scans, while included in the IWOS criteria, were not requested in any of the cases in this study and hence an unknown number of patients may possibly further fulfil the criteria with positive result of these tests.

A subset of patients managed clinically as presumed sarcoid choroiditis did not meet IWOS diagnostic criteria for ocular sarcoidosis. Sub-group analysis revealed reduced anterior segment and vitreous inflammation suggesting disease predominantly confined to the choroidal stroma. Conversely, optic disc nodules/granulomas and/or solitary choroidal nodule—reported in 7–9% of IWOS-concordant ocular sarcoidosis cases—were observed in 29% of IWOS-non-concordant patients. Given the reported high specificity of 97% of these lesions for ocular sarcoidosis, this subgroup would likely have been missed by the 2019 IWOS criteria in the absence of ICGA [10]. These findings should be interpreted cautiously due to the small sample size.

IWOS-non-concordant sarcoid choroiditis patients also showed very low evidence of systemic involvement, with lower proportion of patients having BHL, lymphopaenia, raised serum IL-2R level, and raised serum creatinine level, and no case of hypercalcaemia, abnormal LFTs, positive EBUS/mediastinoscopic lymph node sampling, or pulmonary involvement/systemic syndrome. This, again, may suggest a confined choroidal stromal inflammation with little extension to the more anterior parts of the eye or systemically. In this sense, timeliness of referral in this subgroup of patients may be laxer, especially in cases with negative BHL on either CXR (sensitivity 64%) or CT scan (sensitivity 94%) [21]. Incorporation of ICGA in the assessment of presumed sarcoid choroiditis can add valuable information guiding ophthalmologists towards appropriate ocular diagnosis and management whilst not necessarily putting more pressure on the respiratory clinics, which in many hospitals may still be struggling to cope with workloads following the recent COVID-19 pandemic. Interpretation of this data, however, should be done with caution due to low number of patients in this subgroup.

Limitations of our study include relatively small sample size of patients and retrospective case review methodology. Future studies employing the use of ICGA in ocular sarcoidosis assessment are suggested to be pursued ideally in a prospective manner with larger and more inclusive sample size, with HLA-A29 test considered to definitely exclude Birdshot chorioretinitis.

In summary, patients with presumed sarcoidosis in our cohort were predominantly white and demonstrated higher prevalence of multiple chorioretinal peripheral lesions and optic disc nodules/granuloma and/or solitary choroidal nodule, alongside a lower frequency of bilateral hilar lymphadenopathy (BHL) on chest x-ray (CXR), compared with broader ocular and systemic sarcoidosis populations. Routine incorporation of ICGA into the assessment of suspected ocular sarcoidosis may facilitate identification of this phenotype. In particular, the IWOS-non-concordant subgroup may warrant a less urgent approach to systemic evaluation than IWOS-concordant cases and patient with more typical ocular sarcoidosis presentations.

## Supplementary Information

Below is the link to the electronic supplementary material.

Supplementary Material 1

## Data Availability

All data supporting the findings of this study are available within the paper and its Supplementary Information.
